# SDAV, the Rat Coronavirus—How Much Do We Know about It in the Light of Potential Zoonoses

**DOI:** 10.3390/v13101995

**Published:** 2021-10-04

**Authors:** Michalina Bartak, Anna Słońska, Marcin W Bańbura, Joanna Cymerys

**Affiliations:** Division of Microbiology, Department of Preclinical Sciences, Institute of Veterinary Medicine, Warsaw University of Life Sciences—SGGW, Ciszewskiego 8, 02-786 Warsaw, Poland; anna_slonska@sggw.edu.pl (A.S.); marcin_banbura@sggw.edu.pl (M.W.B.)

**Keywords:** SDAV, rat infections, coronaviruses, zoonoses

## Abstract

Sialodacryoadenitis virus (SDAV) is known to be an etiological agent, causing infections in laboratory rats. Until now, its role has only been considered in studies on respiratory and salivary gland infections. The scant literature data, consisting mainly of papers from the last century, do not sufficiently address the topic of SDAV infections. The ongoing pandemic has demonstrated, once again, the role of the *Coronaviridae* family as extremely dangerous etiological agents of human zoonoses. The ability of coronaviruses to cross the species barrier and change to hosts commonly found in close proximity to humans highlights the need to characterize SDAV infections. The main host of the infection is the rat, as mentioned above. Rats inhabit large urban agglomerations, carrying a vast epidemic threat. Of the 2277 existing rodent species, 217 are reservoirs for 66 zoonotic diseases caused by viruses, bacteria, fungi, and protozoa. This review provides insight into the current state of knowledge of SDAV characteristics and its likely zoonotic potential.

## 1. Introduction

Recently, research interest has turned to animals known to be sources of zoonoses, including primates and other companion animals [1]. The outbreak of the new coronaviral pandemic in 2020 multiplied the quantity of research concerning animals, such as horseshoe bats (*Rhinolophus* spp.), fruit bats (*Rousettus aegyptiacus*), bank voles (*Myodes glareolus*), and raccoons, as well as cats, dogs, ferrets, mink, and other livestock such as pigs and cows. Most of the studies were performed to evaluate if these animals are capable of ACE2 expression, the major SARS-CoV-2 human cell entry receptor [2,3,4,5,6].

The majority of articles have been oriented on bats, but there is a need to focus on rodents, primarily rats. Rodents have been recognized as reservoirs or carriers for several zoonotic viruses, causing, for example, hemorrhagic fever with renal syndrome, Omsk hemorrhagic fever, Apoi virus disease, lymphocytic choriomeningitis virus (LCMV), Western equine encephalitis, and hepatitis E cowpox [7,8]. Notably, *Rattus* spp. thrive in urban areas and are so well-adapted to close cohabitation with people that they are rarely found in habitats devoid of humans. Rats can be found in almost every corner of every city on Earth, and humans are more likely to interact with them than any other wildlife species [9].

Considering this, in the present review, we will describe the current state of knowledge on Sialodacryoadenitis virus (SDAV), its characteristics and potential zoonotic threat.

## 2. Rat Coronaviruses: The Origin

### 2.1. Discovery

The first report on the new member of the coronavirus family appeared in the 1960s, as the laboratory rats had been experiencing destructive sialoadenitis, dacryoadenitis, and transmission disease of the lower respiratory tract [10,11,12,13]. After an outbreak in laboratory rats, possibly antigenically related to mouse hepatitis virus (MHV), agents were found in rat sera by the team of Hartley in 1964 [14,15]. This discovery was confirmed by Jonas et al. in their study of a pathogenic agent, then recognized as virus-like particles in an electron-microscopic examination of the infected salivary glands of rats [10]. Further studies conducted by the team of Parker, led to the isolation of Parker’s rat coronavirus (RCV-P) from the lungs of asymptomatic rats [16]. A second newly recognized strain causing sialoadacryoadenitis (SDA) was also antigenically related to MHV and the rat coronavirus of Parker [17]. Subsequent studies evaluated several other strains such as Japanese isolates, causative agent of rat Sialoadenitis (CARS) [18,19]; and U.S. isolates RCV-BCMM, RCV-W [20], and RCV-NJ [21].

### 2.2. Sialodacryoadenitis Virus: Transmission, Clinical Signs of Infection, and Diagnosis

Experimental studies demonstrated that RCoVs (rat coronaviruses) can remain infectious when dried on solid surfaces [22]. Spread of the infection is relatively easy and can occur by direct contact with infected individuals or by aerosol. There is no evidence for intrauterine transmission. Morbidity frequently reaches 100% among conventionally housed rats [23].

Rat coronaviruses can cause two types of infection: asymptomatic or symptomatic, with tissue tropism to salivary glands, lacrimal glands, Harderian glands, and respiratory epithelium. Moreover, there are two models of infection. The first type develops in breeding colonies (virus present endemically), where an epizootic develops in young non-immune individuals who develop conjunctivitis lasting up to a week [24]. The second type is associated with the sudden onset of episcleritis in naive rats from weaning to adulthood. Within this clinical picture, the symptoms persist for up to 2 weeks and, as with the herpes virus, complications may lead to keratitis and megaloglobus. Other frequently observed signs of SDAV infection include edema of the submaxillary salivary glands, nasal, and ocular discharge (characteristically porphyrin stained), lacrimation, photophobia, corneal opacities, corneal ulcers, and cervical swelling due to inflammation. Moreover, ancillary effects may include transient anorexia and weight loss and disruption of estrus [25,26,27,28,29].

SDAV and other RCoVs are today detectable by immunohistochemical techniques and serological tests, e.g., multiplex fluorescent immunoassay (MFI) with IFA confirmation, ELISA. Prior to seroconversion, especially in the case of an outbreak, histological examination of the Harderian glands and the submaxillary and parotid salivary glands may be necessary [30]. In addition, molecular diagnostic RT-PCR for the M gene (membrane glycoprotein gene), N gene (nucleocapsid gene), and *pol* gene are performed from a fragment of infected tissue, feces, or oral or cage swabs to confirm the initial diagnosis [31,32,33]. Individuals that are diagnosed as positive are quarantined or eliminated. Quarantine also includes animal rooms, which should be disconnected from use for at least 5–8 weeks. Since SDAV is highly contagious, personnel handling quarantined animals may become an important risk factor for transmission, because virus particles can be transmitted on protective clothing [23].

## 3. Characteristics

### 3.1. The General Classification

Coronaviruses, along with toroviruses, roniviruses, arteriviruses, and mesoniviruses, belong to the order *Nidovirales* (‘nido’ means ‘nest’), so named because of the nested subgenomic RNAs generated during the replication cycle [34]. The *Nidovirales* order exhibits enveloped, non-segmented positive-sense RNA viruses. The characteristic features include a highly conserved genomic organization, expression of non-structural protein (NSPs) genes by ribosomal frameshifting, and several unique, non-conventional enzymatic activities encoded within the large replicase. The viruses with the largest capacity to cause epidemics and pandemics among the eight suborders of the order *Nidovirales* are the *Cornidovirineae*. In the *Cornidovirineae* suborder there is one family, *Coronaviridae,* divided into two subfamilies: *Letovirinae* and *Orthocoronavirinae* [35].

**Figure 1:**
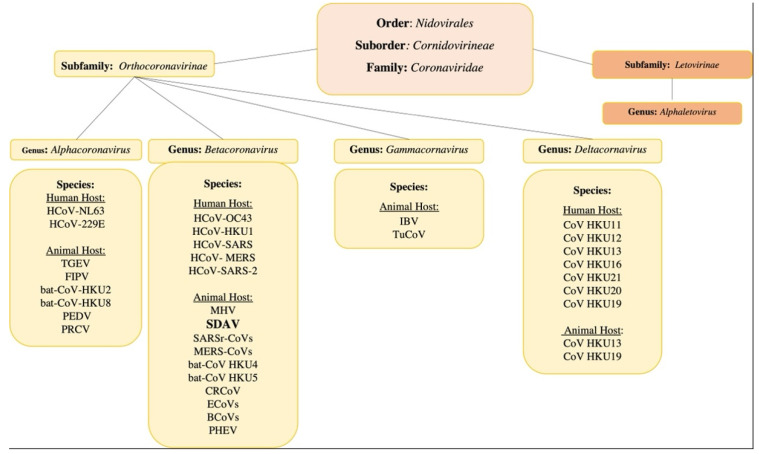
Taxonomy graph of the *Coronaviridae* family. Own work based on ICTV 2020 [36]. Abbreviations: alphacoronaviruses: HCoV-NL63 (human coronavirus NL63), HCoV-229E (human coronavirus 229E), TGEV (transmissible gastroenteritis coronavirus), FIPV (feline infectious peritonitis virus), bat-CoV-HKU2 (Rhinolophus bat coronavirus HKU2), bat-CoV-HKU8 (Rhinolophus bat coronavirus HKU8), PEDV (porcine epidemic diarrheal virus), PRCV (porcine respiratory coronavirus); Betacoronaviruses: HCoV-OC43 (human coronavirus OC43), HCoV-HKU1 (human coronavirus HKU1), SARS (severe acute respiratory syndrome coronavirus), MERS (Middle East respiratory syndrome coronavirus), SARS-CoV-2 (Severe acute respiratory syndrome coronavirus -2), MHV (mouse hepatitis virus), SDAV (sialodacryoadenitis virus), bat-CoV- HKU4 (Tylonycteris bat coronavirus HKU4), bat-CoV-HKU5 (Pipistrellus bat coronavirus HKU5), CRCoV (canine respiratory coronaviruses), ECoVs (equine coronaviruses), BCoVs (bovine coronaviruses), PHEV (porcine haemagglutinating encephalomyelitis virus); Gammacoronaviruses: IBV (infectious bronchitis virus), TuCoV (turkey coronaviruses); Deltacoronaviruses: CoV HKU11 (bulbul CoV HKU11), CoV HKU12 (thrush CoV HKU12), CoV HKU 13 (munia CoV HKU13), CoV HKU16 (white-eye CoV HKU16), CoV HKU19 (night heron CoV HKU19), CoV HKU20 (wigeon CoV HKU20), CoV HKU21 (moorhen CoV HKU21).

The *Coronaviridae* family comprises the largest RNA viruses, in terms of genome length (~30,000 nucleotides) and virion size (spherical, 80–180 nm in diameter). They infect birds and mammals, causing numerous diseases of the respiratory system, nervous system, internal organs, or digestive system. The *Orthocoronavirinae* subfamily is further divided into four genera: Alpha-(14 subgenera and 19 species), Beta-(5 subgenera and 14 species), Delta-(3 subgenera and 7 species), and Gamma-(3 subgenera and 5 species) coronaviruses [36]. From a medical point of view, the most interesting are viruses of the Alpha and Beta genera, which contain human-infecting viruses (alpha-coronaviruses: HCoV-229E and HCoV-NL63, and beta-coronaviruses: HCoV-OC43, HCoV-HKU1, and SARS-CoV, MERS-CoV, and SARS-CoV-2). There are currently seven known human coronaviruses, almost all of them are zoonotic in origin and capable of breaking the species barrier. Of the animal coronaviruses (CoVs), alphacoronaviruses and betacoronaviruses infect only bats and other mammals, while gammacoronaviruses and deltacoronaviruses infect birds, and some of them can also infect marine mammals [37]. Among the most common are PEDV, porcine epidemic diarrhea virus; TGEV, transmissible gastroenteritis coronavirus; FCoV, feline coronavirus; MHV, mouse hepatitis virus; FIPV, feline infectious peritonitis virus; and IBV, infectious bronchitis virus (chicken). CoVs cause a wide variety of diseases in animals, and their ability to be rapidly transmitted among livestock and companion animals led to significant research on these viruses in the second half of the 20th century [38] (Figure 1).

### 3.2. The Rat Coronaviruses

**Figure 2:**
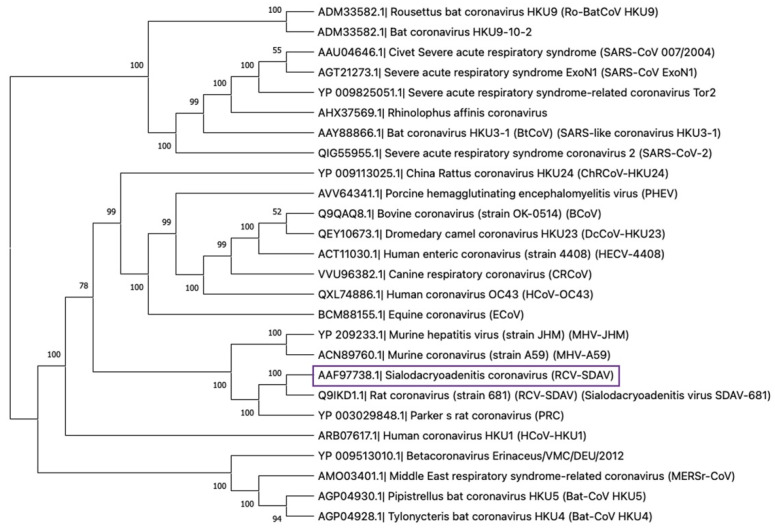
The neighbor-joining tree involves 26 amino acid sequences of spike glycoprotein of betacoronaviruses. The tree was constructed using the p-distance model and 1000 bootstraps in the MEGA X. 10.2.6. The SDAV is indicated in purple.

To date, all known rat coronaviruses belong to the Beta genus. Betacoronaviruses contain five subgenera: *Embecovirus, Hibecovirus, Merbecovirus, Nobecovirus,* and *Sarbecovirus.* Members of the subgenera *Embecovirus* include SDAV, PRCV, RCV-BCMM, RCV-W, RCV-NJ, RCV-CARS, and novel ChRCoV (China Rattus Coronavirus) HKU24 [36,39]. The ChRCoV isolated from Norway rats in China represents the murine origin of *Betacoronavirus* 1. The significance of this discovery lies in the fact that it is a distinct species and not derived from either an avian or bat host, forming the basis of a new lineage of A Beta-CoV (A βCoVs), of which rodents are the principal host. ChRCoV HKU24 represents the murine lineage of Betacoronaviruses 1, with the possibility of interspecies transmission from rodents to other mammals. This transmission occurred before the appearance of the human HCoV coronavirus OC43 in the late 19th century [40]. It is also important to point out that the SDAV and other species of the rat coronaviruses belong to the same genus as the highly epidemic/pandemic and most pathogenic CoVs for humans, such as SARS-CoV, MERS-CoV, SARS-CoV-2, and others [37] (Figure 2).

### 3.3. Virion Structure and Biological Functions of Proteins

**Figure 3:**
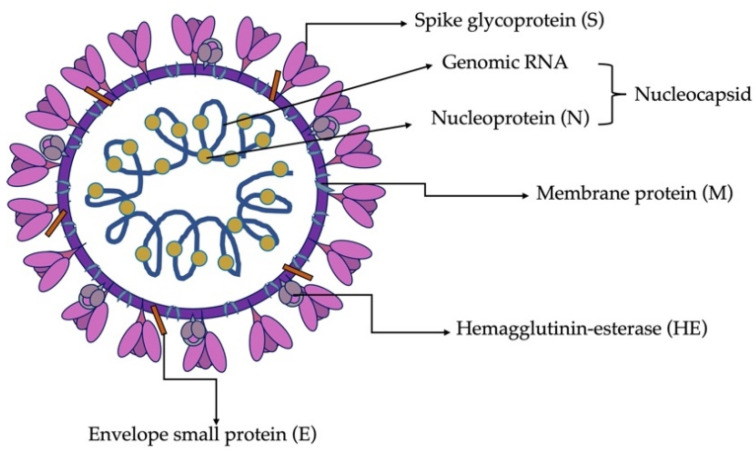
Schematic structure of SDAV virion. Own work based on [42,43,44].

The architecture of a SDAV virion, as in all coronaviruses, is spherical with a diameter ranging from 80 to 180 nm, as confirmed by tomography and cryo-electron microscopy [10]. It consists of a genomic core made up of non-segmented, positive sense, ssRNA stabilized by a nucleocapsid protein and surrounded by a viral membrane envelope. The most characteristic feature of all coronaviruses is the presence of tentacle-shaped spikes, emanating from the surface of the virion and giving it the appearance of a corona, hence the name. CoVs have a helically symmetric nucleocapsid, which is rare among positive sense RNA viruses and much more common in negative sense RNA viruses [41,42,43]. As Barker et al. (1994) and Yoo et al. (2000) described, SDAV contains four major coronaviral structural proteins and one present only in a few species of CoVs. These are the spike (S), membrane (M), envelope (E), nucleocapsid (N) proteins, and hemagglutinin esterase (HE), all of which are encoded at the 3′ end of the viral genome [42,43] (Figure 3).

The homotrimer protein S is the main molecule that binds the virus to the cell surface and is responsible for membrane fusion and entry of the viral genome into the cell. Consequently, it is responsible for SDAV infection, and its gene regions are highly variable and heterogeneous, which determines the change of virulence and tissue tropism. The S protein of SDAV is type I transmembrane N-linked glycosylated protein (149.6 kD), consisting of 1357 amino acids. It is cleaved by host proteases (TMPRESS2, furin) into two subunits: the S1 and S2 [45,46,47,48]. Comparing its sequence to MHV-A59 and MHV-JHM, it has relatively low similarity (76.6%), which comes from additional sequences in the N-terminal half of the S-protein gene of SDAV [43]. Numerous studies have shown that changes in the genes encoding the S protein or related factors can alter CoV virulence, tissue tropism, host range, or host immune response [49].

The second crucial structural protein of SDAV is protein M, a monomer composed of three hydrophobic domains strongly linked to the viral envelope [50]. The M protein of SDAV is 228 aa and 26 kD in size [43]. It has a short N-terminal ectodomain (extracellular domain), modified by glycosylation and C-terminal endodomain situated in the interior of the virion or on the cytoplasmic site of the intracellular membrane [41,51]. The role of protein M is to promote membrane curvature by adapting the membrane region for virion assembly and the uptake of structural protein residues. However, its role in folding virus-like-particles (VLPs), requires the participation and simultaneous expression of protein E. In addition, the M protein participates in the interaction with RNA, by carrying the genomic packaging signal [52].

Small membrane protein (E), made of 88 aa and 10.1 kD, is classified as a homopentamer and is present in small amounts in the virion [53]. It cooperates with protein M during morphogenesis and virion assembly. It also interacts as a viroporin in the host membrane to form pentameric protein-lipid pores that allow ion transport [54].

The nucleocapsid protein (N), the only one present in the helical nucleocapsid, has a size of 454 aa and 49.4 kD and is almost 97% similar to the MHV N protein [55]. Its role is to engage in the replication process by forming homodimers and homo-oligomers, by binding genomic RNA and packaging it, thus forming nucleocapsid. It is also responsible for inhibiting the translation process in the host cell ribosomes. In addition, it collaborates with proteins M and E in the folding and budding process of the newly assembled virions [56]. In other murine coronavirus, such as MHV-JHM, the nucleocapsid protein has been confirmed to be an important enhancer and determinant of neurovirulence [57].

The fifth major structural protein, haemagglutinin esterase (HE), anchored to the viral membrane envelope of SDAV, can bind sialic acid residues on surface glycoproteins and glycolipids. It has acetyl esterase activity and is a homodimer of a size of 439 aa and 49kD [43]. The HE lectin domain contributes to virion attachment and simultaneously enhances sialate-O-acetylesterase activity toward clustered sialoglycotopes. The SDAV HE is highly conserved compared to the MHVs. The HE protein is only present in some embecoviruses (MHV and other rodent CoVs, BCoV, HCoV-OC43, HKU1) [58,59]. It has been postulated that HE is an essential protein for viruses within the betacoronavirus-1 species, including bovine coronavirus (BCoV) and HCoV-OC43 [60,61,62], but for MHV-JHM, MHV-S, and MHV-DVIM it is a non-essential protein [63,64,65]. Its role is more like an additional/supporting binding molecule, an addition to spike protein [66].

### 3.4. Cell Infection and Virus Replication

In rats and mice, SDAV can infect a wide range of cell types, such as the epithelial cells of respiratory airways, mononuclear cells in lymphoid organs, or CNS cells (neurons and astroglia) [19,67,68]. Entry receptor of SDAV has not yet been conclusively confirmed [69]. Unlike closely related viruses (MHV-JHM, MHV-S), SDAV and other RCoVs do not enter cells via CEACAM1 (carcinoembryonic antigen-related cell adhesion molecule 1) receptor or its isoforms [70].

What further distinguishes SDAV, and the other embecoviruses mentioned in Table 1, from other coronaviruses are two surface proteins that allow attachment to the host cell [71,72,73,74,75,76,77,78]. An essential clue in the context of the mechanism of SDAV entry into the cell and binding to the cell receptor was described by Gagneten et al. (1996) [70]. Among other results, the paper showed that only Parker RCV (RCV-P) expresses the haemagglutinin-esterase glycoprotein, which has acetyl-esterase activity. Therefore, SDAV, unlike RCV-P, exhibits the ability to bind to host cell receptors via S glycoprotein. In contrast, RCV-P can bind to the host cell via both haemagglutinin-esterase glycoprotein and spike protein [70]. However, further studies have not been carried out to determine the mechanism of SDAV entry. SDAV probably utilizes the mechanism used by human Betacoronaviruses-1 HKU1 and OC43 and binds via a spike protein to sugar-based receptor-determinates 4-O-acetylated sialic acids (4-O-Ac-Sias) attached as terminal residues to glycan chains on glycoproteins and lipids (forming glycoconjugates) [79]. Whereas HE, a sialate-O-esterase with a specific lectin domain attached to 4-O-Ac-Sia, acts as a receptor-destructive enzyme [44,78]. The hemagglutinin esterase protein promotes virus spread and entry in vivo by facilitating reversible virion attachment to O-acetylated sialic acids [80,81]. In the case of another representative of the embecoviruses, bovine coronavirus, HE activity at the end of replication results in the destruction of intracellular and surface receptors, facilitating the release of progeny virions from the infected cell [44].

After viral S and HE protrusions bind to glycan glycoconjugate receptors on the host cell membrane, fusion with the membrane of endocytic vesicles and virus entry into host cells occurs [49,79]. Most CoVs’ spike proteins are cleaved; however, there are coronaviruses (FIPV, BCoV, MHV-Y, MHV-2) that fuse with cells without cleavage of S protein. In the case of SDAV, S protein undergoes proteolytic cleavage to two subunits: S1 and S2. The interesting fact is that the cleavage recognition sequence (HRARR) is identical to that of MHV-Y, which is not cleaved [43,82]. In fact, the cleavage of the spike (S) protein occurs at two different points of the infection cycle in CoVs. The first, usually induced by furin, results in the separation of the receptor-binding domain (RBD) from the fusion protein S. Following binding of the virion to the receptor, a second proteolytic cut by TMPRRS2 protease, cathepsin, or another occurs. This allows the S2’ fusion peptide to be exposed, thus, it fuses the viral envelope with the cellular membrane, ultimately introducing RNA into the cytoplasm [83]. This process occurs directly on the plasma membrane or via endosomal vesicles and is always triggered by receptor binding [84]. However, this mechanism differs between coronaviruses species and strains and also depends on cell type. The MHV-JHM strain is capable of fusion at neutral pH but was also found in endosomal vesicles (by endosomal pathway) [85]. Recent studies have shown that MHV-JHM uses either the endosomal or the non-endosomal pathway, depending on the cell type. After fusion of the viral envelope with the endosome membrane, the viral genome is released into the cytoplasm. It has been shown that for MHV-4, the fusion is dependent on low pH, which is necessary to mature vesicles to late endosomes and subsequently set up an infection [85,86].

**Figure 4:**
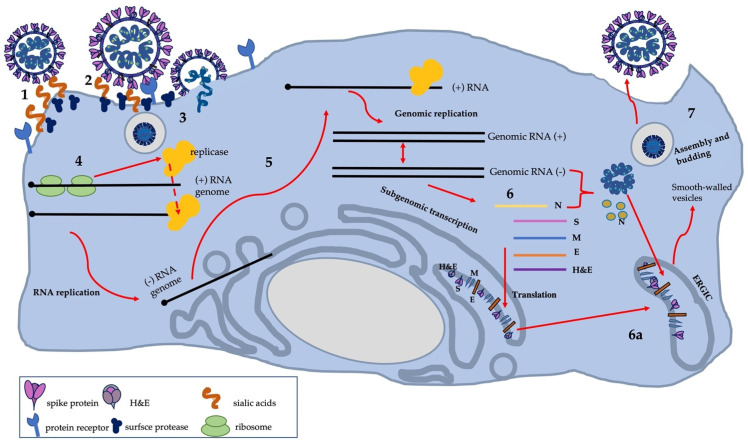
The scheme of SDAV replication: own work based on available knowledge. (1) SDAV HE probably mediates weak interactions with abundant host cell surface sialates, keeping the virus concentrated on cells before connecting S protein with the still unknown cell receptor. (2) S-glycoproteins then bind to protein receptors and are proteolytically activated (by cutting with specific proteases: TMPRESS2 or furin) into two subunits, S1 and S2, to conformations that induce membrane fusion. (3) Virus–cell fusion involves S binding to sialic acids. The likely route of entry into the cell is cell–virus fusion associated with S-protein connections to sialic acids. Connections to host cell receptors may not be required for this process, thus allowing distribution, beginning with the binding of viral protein S (RBD/S1) on the host cell receptor, driving a conformational change in the S2 subunit and facilitating its fusion with the plasma membrane and ultimately the release of viral genome (+ ssRNA) into the cytoplasm. However, the route by which SDAV enters the cell is still unknown and also can be mediated by acidified endosomes. (4) Promptly, the translation produces nonstructural pp1a and pp1ab co-terminal polyproteins (pp1a and pp1ab) that are proteolytically cleaved and assembled into a functional replicase–transcriptase complex: RTC. (5) Viral RNA synthesis produces copies of gRNA, as well as a nested set of sgRNA. This is possible by discontinuous transcription of a negative-sense RNA intermediate. (6) Following replication and sgRNA synthesis, various structural and accessory proteins are translated into the endoplasmic reticulum (ER) and (6a) then assembled as virion in the endoplasmic reticulum-Golgi intermediate component (ERGIC), along with viral genomes encapsulated by N protein budded into membranes of the ERGIC. (7) Then, virions are transported in vesicles and exit cells via exocytosis.

The next step of viral replication is translation of the replicase gene from virion genomic RNA. In the case of SDAV, the complete replicase gene sequence is thought to be as yet undiscovered [43]. However, the region of the replicase gene was described by Stephensen et al., 1999, while developing a consensus PCR assay for coronaviruses [87]. Two overlapping open reading frames (ORF) encode replicase genes: *repl1a* and *rep1b*, which express large polyproteins: pp1a and pp1ab. For these two polyproteins to be expressed, the virus uses a slippery sequence (5′-UUUAAAC-3′) and a pseudoknot RNA, which causes a ribosomal frameshift from *rep1a* to *rep1b* ORF (-1 frameshift). This process lasts until the unwinding of the RNA pseudoknot and the encountering of the *rep1a* stop codon, or pseudoknot blocks the ribosome from further elongation resulting in a stop at the slippery sequence, causing a -1 frameshift before extended translation into *rep1b*, which produced pp1ab polyprotein [88]. These polyproteins pp1a and pp1ab are co-translationally and post-translationally processed into the individual non-structural proteins (nsps). Pp1a and pp1ab contain the nsp 1–11 and 1–16, respectively, forming the viral replication and transcription complex. Proteolytic cleavage of these polyproteins into sixteen nsps is possible by two cysteine papain-like protease (PLP1, PLP2) and chymotrypsin-like protease. These non-structural proteins are then assembled into replicase-transcriptase complex (RTC) and dedicated to viral RNA synthesis [89,90,91]. With the formation of RTC and an environment suitable for RNA synthesis, the process of viral RNA replication and transcription of subgenomic mRNAs begins. The created microenvironment consists of viral replication organelles of characteristic perinuclear double-membrane vesicles (DMVs), convoluted membranes (CMs), and small open double-membrane spherules (DMSs) [69]. The whole replication process is initiated by synthesizing a full-length negative-sense genomic RNA, the template for producing a new positive-sense genomic RNA. Therefore, the generated fragments are then used for translation to produce more nsps and RTCs or packaged into new progeny virions. Notably, the transcription process of coronaviruses is a discontinuous viral transcription that involves the production of a set of nested 3′- and 5′-terminal subgenomic RNAs (sgRNAs) [92]. During negative-strand RNA synthesis, the RTC interrupts transcription against a transcriptional regulatory sequence (TRS) at the 3′ end and restarts upon encountering a TRS-L leader sequence at the 5′ end. The initiated re-synthesis of RNA in the TRS-L region results in the final negative-strand sgRNA formation, by attaching a copy of the negative-strand leader sequence to the nascent RNA strand. These strands are used as templates for the production of the characteristic nested subgenomic positive sense mRNA. The sgmRNA positive sense is successively used for the translation of structural and accessory proteins (spike protein (S), envelope protein (E), membrane protein (M), nucleocapsid protein (N), and hemagglutinin esterase (HE)) [93,94,95]. These proteins are transported to the endoplasmic reticulum and then to the ERGIC compartment (endoplasmic reticulum-Golgi intermediate compartment), where after the entry of viral RNA, the formation of mature virions occurs. The virions are transported in secretory vesicles to the vicinity of the cell membrane, where their exocytosis occurs, as with most coronaviruses (Figure 4). For SDAV, the mechanism of egress has not been clearly described, but for MHV, it is known to utilize the lysosomal trafficking pathway with antigen presentation. Intriguingly, some of the produced and unused S-proteins are transported to the surface of the cell membrane, allowing the connection of several neighboring cells, and thus easier propagation of the infection without going into the extracellular space and alarming the immune system [96,97,98].

### 3.5. Genomic Structure and Gene Functions

**Figure 5:**
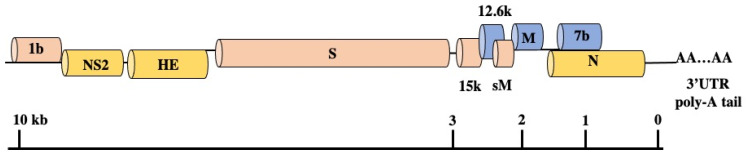
Genome structure and ORFs in the 3′ end 9.8 kb of SDAV viral genome. Own work based on [43].

Until the 2000s, little was known about SDAV structural proteins. The only knowledge available was based on serological tests, without a concrete distinction between specific proteins. However, the first trial was attempted in 1993 by Kunita et al., on obtaining the complete nucleotide sequence of the N gene of SDAV-681 and establishing its similarity to other coronaviruses [55]. Additionally, it was known that antibodies specific for the structural proteins (spike protein, nucleocapsid protein, and membrane protein) of MHV, could recognize SDAV and PRCV using the immunoblot technique [42]. The next attempt was made in 2000 to characterize the remaining structural proteins and genome structure of SDAV by the team of Yoo et al. [43]. They sequenced the 3′ terminal 9.8 kb of the SDAV genomic RNA. During the research, using specially designed primers based on several coronaviral sequences, it was possible to generate seven cDNA fragments by RT-PCR cDNA cloning, which represented the 3′ terminal of the genome [43]. By amplifying various fragments, they confirmed the coding sequences for S, M, and N structural proteins and non-structural proteins, NS2 and HE gene in nine major open reading frames (ORFs), as is shown in the Figure 5. The coding sequence for 12.6k (ORF5a, non-structural protein), M (membrane-associated protein), and 7b (ORF7b, small internal ORF) is present in the +1 frame. The encoding sequence for polymerase 1b is present in the +2 frame, as well as genes for 15k (15k nonstructural protein), S (spike protein), and sM (small membrane protein). The other sequence for NS2 (nonstructural protein NS2), HE (hemagglutinin-esterase) and N (nucleocapsid protein) is placed in +3 frame. The NS2 gene absent in the MHV-JHM Wb1 variant is present in SDAV. In contrast to the HE gene region of MHV-JHM; MHV-4; MHV-DIVM, the SDAV HE nucleotides are highly conserved and exhibit low (58%) homology with other coronaviruses (BCV or OC43) but maintain a relatively high MHV amino acid identity (91%). In contrast to a previous statement in research by Gangneten et al., 1990, the HE protein is expressed by SDAV and maintained as in BCV, HCoV OC43, MHV-JHM, MHV-4, MHV-DVIM, and human Influenza type C [70]. Moreover, in small internal ORF (ORF7b) and the untranslated 3′ region (UTR), 298 encoding amino acids with a polyadenylated tail have been recognized. Additionally, each protein gene identified a short intergenic consensus sequence [43].

In 2002, the method of detecting rodent coronaviruses, including SDAV, was improved by fluorogenic RT-PCR, known as Real-Time PCR/TaqMan PCR. Besselsen and colleagues designed a novel reverse transcriptase PCR procedure using an internal fluorogenic hybridization probe to detect MHV and RCVs. The reaction utilized selected fragments from a genome segment of M protein highly conserved among all coronaviruses [33]. This method improved detection and in vitro quantitative analysis assays for SDAV research; previously invented by Yoo et al., 2000 [43].

### 3.6. SDAV Propagation and Role of In Vitro Studies

Until recently, SDAV had been mainly used in the evaluation of alveolar epithelial cell response to infection. The team of Miura et al. (2007) detected the ability of uninfected alveolar type I epithelial cells to produce chemokines as a response to IL-1 produced by SDAV/RCoV-P-infected cells and leading to pulmonary inflammation [100]. Further research by Funk et al. (2009) provided insight into SDAV-infection induced chemokine expression in alveolar type I, type II, and airway epithelial cells. They confirmed the importance of SDAV as a model for the early events of innate immune response to respiratory CoVs infections of natural hosts, and they also detected that type I epithelial cells stand as a primary target of SDAV infection and the crucial role of CXC chemokine expression following infection and innate immune response cell influx [68].

As currently known, SDAV in vitro propagation is possible in established cell lines, such as murine fibroblasts L-2(Percy) cells, subclone L2P-41.a, LBC cells, RCV-9, and in primary rat kidney cell cultures (RKCC). In other frequently used cell lines (VERO, Hep-2, NTCT 1469, BHK-21), infection is not possible [15,28,70,101,102,103,104]. For purification and plaque assay, L-2cells, L2P-41.a, and LBC are often used [100,101]. The virus samples can be stored at −60 °C for 7 years, but according to research by Jacoby et al. (1975), they can become less infectious when stored at −20 °C [11,105].

## 4. Conclusions

Despite many commonalities with zoonotic coronaviruses of the genus Betacoronaviruses, there is a notable lack of interest in rat coronavirus SDAV. When considering SDAV in the context of a potential zoonosis, and not just an enzootic laboratory threat, the insufficient knowledge of the entire replication cycle after entry into the organism must be taken into consideration. Information on the entry receptor and the surface interaction with the host cell is still unclear. Indeed, on the basis of the information presented in this review, it is tempting to speculate that most of the processes are identical or very similar to other representatives of embecoronaviruses and betacoronaviruses. However, the high capacity of this family for transient mutations in the hypervariable region of the main structural glycoprotein, the S-spike protein, may lead to another crossing of the inter-species barrier, as happened in 2019 in Wuhan with Sars-CoV-2 [38]. Information about the possible SDAV infection of mice was confirmed in the 1970s by the teams of Parker (1970) and Bhatt (1972) [16,17]. The growing number of new coronaviruses recently is worrying, due to the close relationship between coronaviruses and distantly related animals. Such interspecies jumps can cause uncontrolled outbreaks of zoonoses. Examples include: (i) FCoV and CCoV in the alphacoronaviruses group; (ii) MHV and the aforementioned SDAV; other RCoVs or HCoV -OC43, BCoV and PHEV; SARS -CoV-1, SARS-COV-2, and MERS-CoV in the betacoronaviruses group; (iii) IBV, TCoV, Asian leopard cat coronavirus and new avian coronaviruses in the gammacoronaviruses group [38,106,107].

Symptomatic SDAV infection is currently relatively easy to control under laboratory conditions [108]. When infection occurs and it is confirmed by PCR tests, infected animals can be eliminated. However, mild or asymptomatic infections can become a problem, providing ideal conditions for the virus to mutate. Poorly maintained hygienic conditions in mouse or rat cages and inadequate management of potentially infectious waste could contribute significantly to undesired SDAV transmission. This problem was highlighted in a work of de Bruin et al. (2016). A systematic review revealed that through the transfer of soiled bedding (mainly spread by the oral and fecal route), infection is effectively transmitted in cases of mouse hepatitis virus (MHV), mouse parvovirus (MPV), and Theiler’s mouse encephalomyelitis virus (TMEV). Unfortunately, in the context of SDAV infection or other infectious agents (minute virus of mice (MVM), Sendai virus (SeV), murine rotavirus (EDIM)), insufficient data are available for evaluation [108].

At this time, only studies on respiratory infections caused by SDAV have been described. More studies should be conducted to identify the mechanism of virus–cell interaction on different cell models. In vitro CNS-SDAV model studies are still lacking, and based on recent studies concerning SARS-CoV-2, it appears that this virus (SDAV) may cause significant changes in the central nervous system, as in other CoVs [18,67,109].

Based on actual knowledge and general properties of coronaviruses a main question can be asked. If SDAV a possible threat to humans? Considering the algorithm and risk of zoonotic potential designed by Palmer et al. in 2005, there are three crucial stages in the transmission of a pathogen, which comprise the levels of potential risk [110]. The risk of cross species transmission and exposure of humans to an infected host is relatively substantial. The high capacity of coronaviruses to mutate within the genome, especially the S-protein subunit (spike), leads to rapid adaptation and transmission among multiple hosts. The facts of the facilitated potential of coronaviruses to cross the inter-species barrier and being a host commonly found near humans highlights the need for further research into the characterization of SDAV infections.

## Figures and Tables

**Table 1:** Examples of viruses expressing the O-acetylesterases. Based on [44,99].

Family	Genus	Species	Substrate *
*Coronaviridae*	Coronavirus	Human coronavirus OC43	Neu5,9Ac_2_
		Bovine coronavirus	
		Hemagglutinating encephalomyelitis virus	
		Mouse hepatitis virus	Neu4,5Ac_2_
		Puffinosis coronavirus	Neu4,5Ac_2_
		Sialodacryoadenitis virus	Neu4,5Ac_2_
	Torovirus	Porcine torovirus	n.d.
		Bovine torovirus	
*Orthomyxoviridae*	Isavirus	Infectious salmon anemia virus	Neu4,5Ac_2_
	Influenza C virus	Influenza C virus	Neu5,9Ac_2_ (α2-8-linked, GL)
	Influenza D virus	Influenza D virus	Neu5,9Ac_2_

* Abbrevations: Neu5,9Ac2 (*5-N-acetyl-9-O-acetyl neuraminic acid*); Neu4,5Ac2 (N-Acetyl-4-O-acetylneuraminic acid); *GL* (*glycolipids*).
